# Policy Series: From Research to Action: Advancing Social Connection and Engagement for Older Adults

**DOI:** 10.1093/geroni/igaf122.1042

**Published:** 2025-12-31

**Authors:** Jillian Racoosin Kornmeier

## Abstract

Social isolation and loneliness (SIL) pose serious risks to the physical, cognitive, and mental well-being of older adults. While social connection is a critical determinant of health, older adults often encounter unique challenges—such as living alone, retirement, disability or illness, and reduced mobility—that heighten their vulnerability to SIL. Therefore, ensuring they have the ­necessary support to stay socially engaged is essential. In this symposium, experts in research, practice, policy, and philanthropy will explore the multifaceted causes of SIL in older adults, analyzing how individual, community, and systemic factors intersect. They will also examine evidence-based strategies and existing practices that promote social engagement, drawing from the Foundation for Social Connection’s Systems Of Cross-sector Integration and Action across the Lifespan (SOCIAL) Framework reports on the Built Environment, Arts & Culture, and Nutrition sectors. Additionally, they will discuss key legislative efforts, including the Older Americans Act (OAA) and the Safeguarding Elderly Needs through Innovation and Occupational Resources (SENIOR) Act of 2024. Finally, they will highlight research gaps and future directions to advance solutions that foster social connection and promote healthy aging. This will include insights from the philanthropic sector into how funders can drive impact—highlighting opportunities for investment, cross-sector collaboration, and targeted funding approaches that support sustainable solutions. Attendees will leave this session equipped with practical skills, actionable strategies, and a clear understanding of how to advocate for and enact meaningful change in their communities. Loneliness and Social Isolation Interest Group Sponsored Symposium

